# Characterization of Electronic Health Record Use Outside Scheduled Clinic Hours Among Primary Care Pediatricians: Retrospective Descriptive Task Analysis of Electronic Health Record Access Log Data

**DOI:** 10.2196/34787

**Published:** 2022-05-12

**Authors:** Selasi Attipoe, Jeffrey Hoffman, Steve Rust, Yungui Huang, John A Barnard, Sharon Schweikhart, Jennifer L Hefner, Daniel M Walker, Simon Linwood

**Affiliations:** 1 Division of Health Services Management and Policy College of Public Health The Ohio State University Columbus, OH United States; 2 Division of Clinical Informatics Nationwide Children's Hospital Columbus, OH United States; 3 Department of Pediatrics The Ohio State University College of Medicine Columbus, OH United States; 4 The Abigail Wexner Research Institute Nationwide Children's Hospital Columbus, OH United States; 5 Department of Family and Community Medicine College of Medicine The Ohio State University Columbus, OH United States; 6 The Center for the Advancement of Team Science, Analytics, and Systems Thinking College of Medicine The Ohio State University Columbus, OH United States

**Keywords:** electronic health records, access log analysis, pediatrics, primary care physicians, work outside work, work outside scheduled clinic hours

## Abstract

**Background:**

Many of the benefits of electronic health records (EHRs) have not been achieved at expected levels because of a variety of unintended negative consequences such as documentation burden. Previous studies have characterized EHR use during and outside work hours, with many reporting that physicians spend considerable time on documentation-related tasks. These studies characterized EHR use during and outside work hours using clock time versus actual physician clinic schedules to define the outside work time.

**Objective:**

This study aimed to characterize EHR work outside scheduled clinic hours among primary care pediatricians using a retrospective descriptive task analysis of EHR access log data and actual physician clinic schedules to define work time.

**Methods:**

We conducted a retrospective, exploratory, descriptive task analysis of EHR access log data from primary care pediatricians in September 2019 at a large Midwestern pediatric health center to quantify and identify actions completed outside scheduled clinic hours. Mixed-effects statistical modeling was used to investigate the effects of age, sex, clinical full-time equivalent status, and EHR work during scheduled clinic hours on the use of EHRs outside scheduled clinic hours.

**Results:**

Primary care pediatricians (n=56) in this study generated 1,523,872 access log data points (across 1069 physician workdays) and spent an average of 4.4 (SD 2.0) hours and 0.8 (SD 0.8) hours per physician per workday engaged in EHRs during and outside scheduled clinic hours, respectively. Approximately three-quarters of the time working in EHR during or outside scheduled clinic hours was spent reviewing data and reports. Mixed-effects regression revealed no associations of age, sex, or clinical full-time equivalent status with EHR use during or outside scheduled clinic hours.

**Conclusions:**

For every hour primary care pediatricians spent engaged with the EHR during scheduled clinic hours, they spent approximately 10 minutes interacting with the EHR outside scheduled clinic hours. Most of their time (during and outside scheduled clinic hours) was spent reviewing data, records, and other information in EHR.

## Introduction

Current research suggests that the proliferation of electronic health records (EHRs) has contributed to the increased time physicians spend interacting with computers, often at the expense of direct patient care [1-6]. Prior research has shown that physicians in the United States spend 1 to 2 additional hours completing EHR-related tasks for every hour they spend with patients [7]. Other research on this topic suggests that physicians spend approximately half their workdays on EHRs [8]. This EHR documentation burden was predicted in a systematic review published in 2005 by Canadian researchers, warning that the goal of decreased documentation time with the adoption of EHRs will likely not be realized, particularly among physicians [9].

The increased workload associated with EHR tasks has resulted in many physicians completing their EHR-related tasks during nonwork hours (eg, at night, on weekends, and during vacation time) [7,10,11]. Prior research suggests that physicians spend 90 minutes each day on EHRs outside their normal work hours. A study reported that even among physicians reporting EHR proficiency, more than half (56%) reported time spent at home on EHR-related work was *excessive* or *moderately high*, with less than one-quarter reporting sufficient time for documentation during work hours [12]. In another study, more than one-third of physicians self-reported working outside work hours, with approximately 60% of that time spent using EHRs [5]. A third study reported that of the 6 hours that clinicians spent on EHRs per weekday, 24% of this time was outside work hours [8].

Previous studies have quantified EHR work during and outside work hours [1,4-6,8,13-18] using predetermined times as their definition of work hours. Using the same approach, others have assessed the types of actions completed in EHR during these periods and the time allocated to these actions [8,15]. For instance, clerical and administrative actions (eg, documentation, order entry, billing and coding, and system security) accounted for almost half of the EHR actions (44%), and inbox management accounted for another one-quarter (24%) of that time [8].

The aim of our study is to characterize EHR work outside scheduled clinic hours among primary care pediatricians. The study design, using a retrospective descriptive task analysis of EHR access log data, extends the prior literature by identifying specific actions that are frequently completed outside work hours using physician schedules rather than fixed clock times to define outside work hours. Focusing on schedules instead of clock time allows us to produce more accurate estimates of time spent on the EHR outside of the actual scheduled clinic hours, as physician work schedules can be variable and include evenings and weekends. To our knowledge, no study thus far has used individual physician schedules to classify time spent into work and nonwork hours, which is a critical addition to the dialog and research on EHR-related documentation burden.

## Methods

### Setting

This study used a retrospective analysis of EHR access log data from primary care pediatricians at the Nationwide Children’s Hospital (NCH), a large, free-standing US children’s hospital that uses the Epic EHR (Epic Systems Corporation). All physicians who, in September 2019, generated primary care relative value units (RVUs), a measure of billable service volume and complexity, were included in the study. The use of EHR audit log data collected over a 1-month time frame is recommended because of the amount of work required to collect and clean a larger data set and the potential for shorter periods to better expose anomalies because of events such as vacations and changes in staffing [19]. Pediatricians generating non–primary care RVUs such as in inpatient or urgent care settings were omitted. All the access log data of pediatricians who met the inclusion and exclusion criteria were included in the study.

### Ethics Approval

This study was approved by the institutional review boards of the NCH (protocol number IRB1800261) and Ohio State University (rotocol number 2019N0042).

### Data Acquisition and Preparation

**Figure 1:**
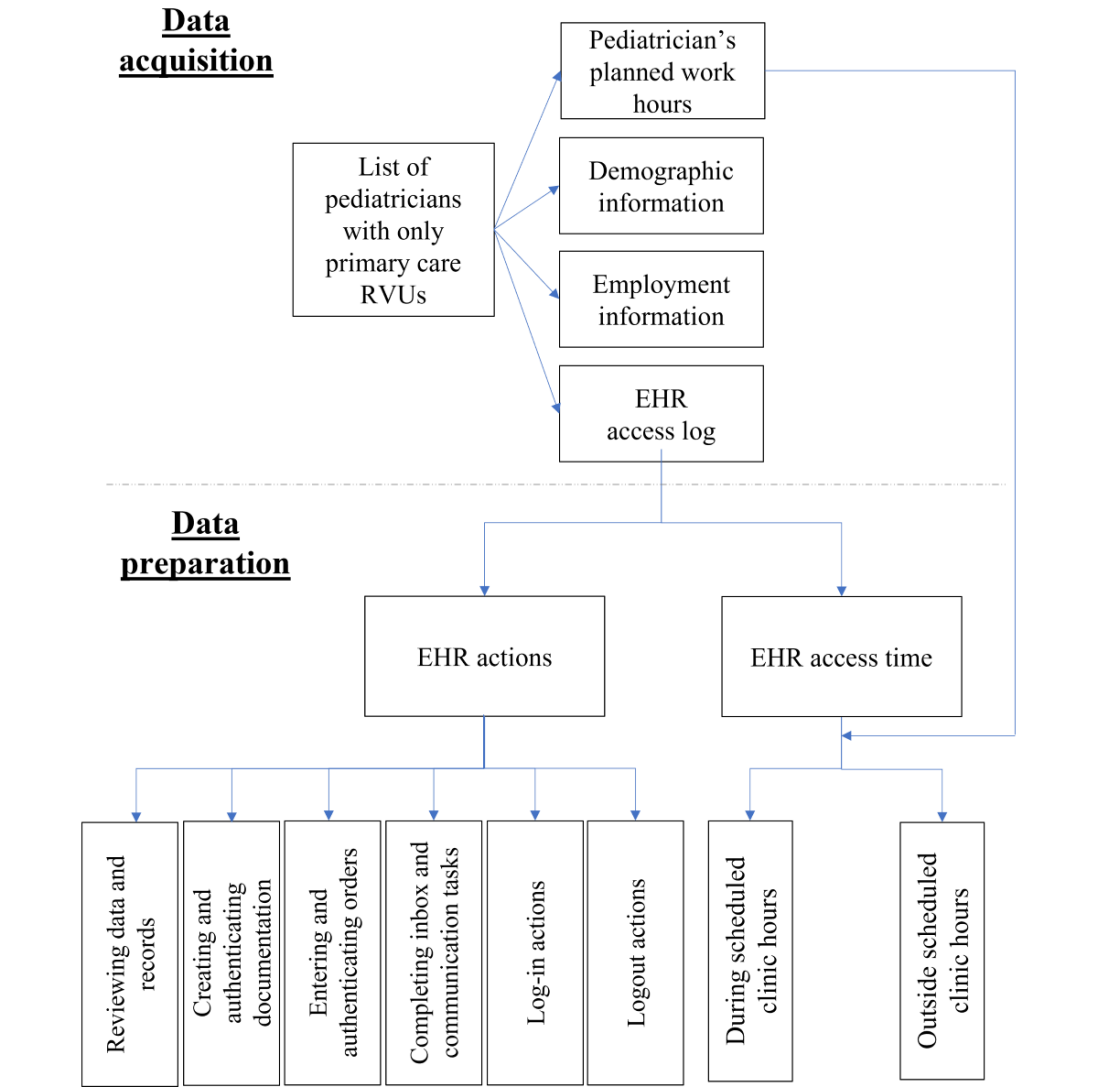
Flow chart of data acquisition and data preparation. EHR: electronic health record; RVU: relative value unit.

Clinical, billing, scheduling, and EHR use data were extracted from the local Epic EHR and other administrative sources into a separate database for analysis (Figure 1). The data included pediatricians’ planned clinic hours, patient appointments, demographic information (eg, age and sex), employment information (length of hospital service, physicians’ total workload or full-time equivalent [FTE] status, and physicians’ clinical workload or clinical FTE [cFTE] status), and EHR access log entries. The *EHR access log* captures discrete time-stamped actions associated with provider navigation and use of the EHR [15,20]. It captures providers’ direct interactions with the EHR system, such as log-in, logout, chart review activity, clinical documentation, and ordering actions [15,20]. Log files also record information such as the user, the time of access, the device from which the EHR was accessed, and the portion of the EHR system that was accessed [15].

The primary variables for our analysis were the *EHR actions* and *access time* extracted from the EHR access log files. *EHR actions* refer to events or movements recorded in the EHR system through mouse clicks and scrolling. These actions were grouped into 6 meaningful action categories (4 clinical and 2 general categories) using an iterative process in which the primary researcher (SA) worked with a clinical informatics physician fellow under the supervision of the NCH Chief Medical Information Officer (JH) to review various actions and associated categories. This process resulted in the identification of four clinical action categories (reviewing data and reports, creating and authenticating documentation, entering and authenticating orders, and completing inbox and communication tasks) and two general action categories (log-in and logout activities).

*EHR access time* (ie, duration or elapsed time) refers to the time spent in the EHR or the time spent completing actions in the EHR. Access time was estimated using a previously validated algorithm used by Arndt et al [8]. Access time was defined as the time between each activity log entry and the next log entry for a given user. The total access time was calculated for all EHR actions for each physician and then decomposed into two mutually exclusive time segments: (1) during scheduled clinic hours and (2) outside scheduled clinic hours.

*EHR work during scheduled clinic hours* was defined as EHR work that occurred during the period 30 minutes before to 30 minutes after scheduled patient visits for each physician each day. Similarly, *EHR work outside of scheduled clinic hours* was defined as work completed outside of the *work hours* period. A margin of 30 minutes was added to each physician’s scheduled clinic hours to capture preparatory actions or closing actions for a set of consecutive patient visits. Finally, we identified and examined high users of EHR outside scheduled clinic hours to determine unique patterns of use.

### Data Analysis

Descriptive task analysis was used to quantify and identify patterns of EHR work completed outside the scheduled clinic hours. All actions spanning >15 minutes were removed to omit occurrences of idle time. This cutoff was determined after careful examination of the data, sensitivity analyses, discussions with the Chief Medical Information Officer (JH), and the acknowledgment that, in practice, a single action in the EHR is typically not >15 minutes. Descriptive statistics (using demographic data) were calculated for the overall physician group. Categorical variables are reported as frequencies and percentages of the total. Continuous variables are summarized as mean and SD. The overall *EHR access time* for each physician was determined by averaging the amount of time spent during and outside the scheduled clinic hours each day across the study month. The overall time and the proportion of time spent on the *actions* completed in the EHR were examined by calculating the time *spent* per physician per workday. Administrative time (ie, time allotted within clinical schedules to complete clinical notes, inbox messages, and other administrative duties related to patient care) was calculated and reported by dividing the total number of hours of administrative time by the total number of physician workdays. The total number of administrative hours was estimated to be approximately 11% of the nominal clinical hours during the 4-week study period. The frequency (or number) and duration of EHR actions were examined to determine which actions were consistently completed outside scheduled clinic hours and whether any patterns emerged.

Regression analyses were also conducted to determine relationships between certain explanatory variables and variations in EHR use. For these analyses, the main outcome variables were the duration of EHR use both during and outside scheduled clinic hours and total EHR use. Mixed-effects statistical modeling was performed using daily and weekly aggregated data to assess the fixed effects of physician age, sex, and clinical FTE status on EHR use and estimate the magnitude of random effects because of variations among providers and temporal differences affecting all providers daily and weekly. The distributions of the outcome variables were analyzed to assess the normality assumption and determine whether a transformation was needed. All data were managed and analyzed using Microsoft Excel (version 16.0.4266) and R (version 3.5.2; R Foundation for Statistical Computing).

## Results

### User Statistics

There were 62 (n=14, 23% male and n=48, 77% female) pediatricians identified as working in the Division of Primary Care Pediatrics who generated primary care RVUs during September 2019, of whom 4 (6%) were excluded because they were employed on a contingency status, 1 (2%) was excluded because she had zero cFTE status, and 1 (2%) was excluded because she did not see patients during the study period. The 56 pediatricians included in the study (n=12, 21% male and n=44, 79% female) generated 1,523,872 EHR access log data points (across 1069 physician workdays). Of the 56 pediatricians, 49 (86%) used EHR outside the scheduled clinic hours. The descriptive statistics are presented in Table 1. The sample group comprised pediatricians aged 30 to 69 (mean 45.6, SD 9.9) years, with an average length of hospital service of 10.1 (SD 7.6) years (range 4 months to 33 years). The average FTE and cFTE statuses were 0.8 (SD 0.2) and 0.5 (SD 0.2), respectively.

**Table 1:** Descriptive statistics (N=56).

Characteristics	Values, mean (SD; range)
Age (years)	45.6 (9.9; 30-69)
Length of hospital service (years)	10.1 (7.6; 0.3-46)
Full-time equivalent status	0.8 (0.2; 0.5-1.0)
Clinical full-time equivalent status	0.5 (0.2; 0.5-0.9)
EHR^a^ work during scheduled clinic hours (hours per physician per workday)	4.4 (2.0; 0.7-8.2)
EHR work outside scheduled clinic hours (hours per physician per workday)	0.8 (0.8; 0-3.2)

^a^EHR: electronic health record.

### EHR Access Time

**Figure 2:**
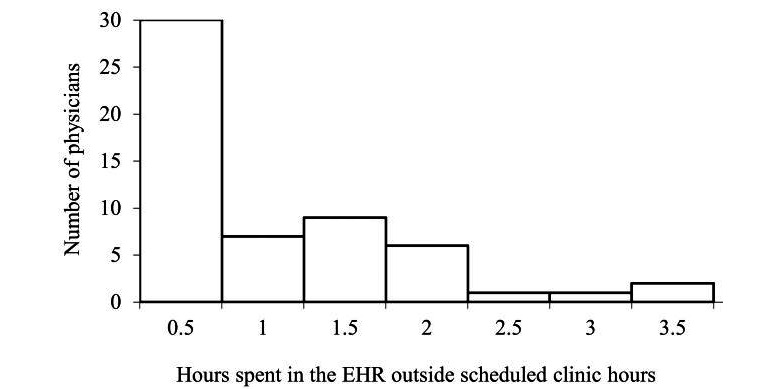
Histogram of average time spent in the electronic health record (EHR) by each physician outside scheduled clinic hours.

The pediatricians in this study had an average of 6 hours of scheduled work time, excluding administrative time. They spent approximately 4.4 (median 4.3) hours per workday interacting with the EHR during scheduled clinic hours and approximately 0.8 (median 0.4) hours per workday outside scheduled clinic hours. On average, the available administrative time was 0.5 hours per workday. EHR use ranged between 0.7 and 8.2 hours during scheduled clinic hours and between 0 and 3.2 hours outside of scheduled clinic hours. When physicians used the EHR outside of scheduled clinic hours, they typically did so in the evenings and on weekends. Figure 2 presents a histogram of the average time spent in the EHR by each physician outside the scheduled clinic hours.

### EHR Action Categories

#### Overview

A total of 290 unique EHR actions were identified, and each action was classified into an EHR action category. Of the 290 EHR actions, 161 (55.5%) were classified as reviewing patient charts, 64 (22.1%) as creating and authenticating documentation, 34 (11.7%) as completing inbox and communication tasks, 19 (6.6%) as entering and authenticating orders, and 12 (4.1%) as completing log-in and logout activities.

#### Action Frequencies and Duration by EHR Action Categories

Table 2 presents an overview of the time spent on EHRs per physician per workday grouped by the EHR action category. Pediatricians spent approximately 73% (3.72/5.12) of their time reviewing data and reports, 7% (0.33/5.12) creating and authenticating documentation, 12% (0.60/5.12) completing inbox and communication tasks, 3% (0.13/5.12) entering and authenticating orders, and 6% (0.33/5.12) engaging in log-in and logout activities. For order entry, only 8% (0.01/0.13) of the work was completed outside scheduled clinic hours, whereas for the other 3 clinical categories, 13% (0.08/0.60) to 16% (0.59/3.72) of the work was completed outside scheduled clinic hours.

**Table 2:** Time spent per physician per workday by action category.

	Hours spent per physician per workday, n (%)		
	During scheduled clinic hours	Outside scheduled clinic hours	Total
Reviewing data and reports	3.13 (72)	0.59 (78)	3.72 (73)
Creating and authenticating documentation	0.28 (7)	0.05 (7)	0.33 (7)
Completing inbox and communication tasks	0.52 (12)	0.08 (11)	0.60 (12)
Entering and authenticating orders	0.12 (3)	0.01 (2)	0.13 (3)
Log-in actions	0.03 (1)	0.01 (1)	0.04 (1)
Logout actions	0.28 (6)	0.02 (2)	0.29 (6)
Total	4.35 (100)	0.76 (100)	5.12 (100)

#### Top 3 Most Frequent Actions by EHR Action Category

Approximately 93.1% (270/290) of EHR actions were completed outside the scheduled clinic hours per physician per workday. Of these 270 actions, the 3 most frequent specific actions completed outside scheduled clinic hours within the 4 clinical action categories accounted for 74 (27.4%) actions and 25 minutes per physician per workday (Table 3). For *chart review,* the most frequent EHR action outside scheduled clinic hours was viewing patient data, which occurred 28 times and over 13 minutes per physician per workday. This trend was similar for EHR use during scheduled clinic hours. For *documentation*, the 2 most frequent activities outside scheduled clinic hours were the use of visit documentation templates (occurring 15 times over 1 minute per physician per workday) and the signing of clinical notes (occurring 2 times over 0.4 minutes per physician per workday). During scheduled clinic hours, the use of visit documentation templates was the most frequent activity; however, the second most frequent activity was the modification of clinical diagnoses. For *inbox and communication*, viewing inbox messages was the most frequent EHR action outside the scheduled clinic hours. This action occurred approximately 8 times over 2 minutes per physician per workday. However, during scheduled clinic hours, the most frequent EHR action in this category was the creation of inbox messages. For *order entry*, the most frequent EHR action outside scheduled clinic hours was the use of outpatient order sets, which occurred 3 times over 0.2 minutes per physician per workday. This trend was similar during the scheduled clinic hours.

**Table 3:** Top 3 most frequent actions completed outside scheduled clinic hours in the EHR^a^ per physician per workday by EHR action category.

		Frequency per physician per workday	Total minutes spent per physician per workday
**Reviewing data and reports**			
	Patient data viewed	28	12.7
	Encounter data viewed	4	3.5
	Clinical notes viewed	4	3.2
**Creating and authenticating documentation**			
	Visit documentation template used	15	1.1
	Clinical note signed	2	0.4
	Encounter diagnoses entered	2	0.3
**Completing inbox and communication tasks**			
	Inbox message viewed	8	2.2
	Inbox message created	3	0.7
	Inbox folder loaded	3	0.7
**Entering and authenticating orders**			
	Outpatient order sets used	3	0.2
	Order list changed	1	0.2
	Length of stay entered	1	0.2
Total		74	25.4

^a^EHR: electronic health record.

#### High Outside Scheduled Clinic Hours EHR Users

EHR use by physicians who spent >1.5 hours per workday outside scheduled clinic hours (10/56, 18%) was further examined to determine if there were additional insights that could be gained from pediatricians who use the EHR more outside scheduled clinic hours. Together, these physicians generated a total of 212 physician workdays, spent an average of 2.2 hours per physician per workday in the EHR outside scheduled clinic hours, and exhibited similar trends (in terms of the most frequent activities completed in the EHR) to those of the entire group.

### Factors Associated With EHR Use

Mixed-effects models revealed no significant associations of age, sex, and cFTE status with EHR use during or outside scheduled clinic hours (Table 4).

**Table 4:** Mixed regression models.

Models			EHR^a^ work during scheduled clinic hours		EHR work outside scheduled clinic hours		Total EHR use
**Fixed effects, coefficients (SE)**							
	EHR work during scheduled clinic hours (minutes)	N/A^b^		−0.18 (0.02)		N/A	
	Age (years)	−0.06 (0.02)		−0.004 (0.01)		−0.05 (0.02)	
	Gender	0.86 (0.50)		−0.80 (0.29)		−0.14 (0.43)	
	cFTE^c^ status	3.63 (0.92)		0.67 (0.54)		3.63 (0.79)	
	Constant	3.00 (1.34)		2.23 (0.76)		4.67 (1.18)	
**Random effects, variance (SD)**							
	Day	3.45 (1.86)		0.06 (0.24)		3.74 (1.93)	
	Provider	1.91 (1.38)		0.63 (0.80)		1. 35 (1.61)	
**Model fitness (*R^2^*; %)**							
	Fixed effects	10.0		9.9		7.8	
	Random effects	41.4		53.4		41.5	
	Total	51.4		63.7		49.3	

^a^EHR: electronic health record.

^b^N/A: not applicable.

^c^cFTE: clinical full-time equivalent.

## Discussion

### Principal Findings

In this study, we quantified and characterized EHR work outside scheduled clinic hours and found that pediatricians spent approximately 0.8 hours per physician per workday completing work in the EHR outside of scheduled clinic hours. The time spent using the EHR outside scheduled clinic hours accounted for approximately 15% of the total daily EHR time (ie, 5 hours per physician per workday). Specifically, outside scheduled clinic hours (ie, 0.76 hours per physician per workday), pediatricians spent 78% of their time (ie, 0.59 hours per physician per workday) reviewing data and reports, 11% (ie, 0.08 hours per physician per workday) completing inbox and communication tasks, 8% (ie, 0.06 hours per physician per workday) documenting and completing orders, and 3% (ie, 0.03 hours per physician per workday) engaging in log-in and logout activities. This distribution across action categories was similar to the distribution of actions during scheduled clinic hours.

### Comparison With Prior Work

The *proportion of total time spent in the EHR outside work hours* in this study (0.76/5.12, 15%) was lower than that reported by Arndt et al (24%) [8], Rotenstein et al (25% and 26%) [14,18], and Holmgren et al (30%) [13] and higher than that reported by Overhage and Johnson (12%) [21] and Holmgren et al (13%) [17]. Each of these alternative estimates characterized EHR use outside work hours using a predefined clock time, whereas this study used actual physician schedules. The methodology used in this study is arguably superior because of the granular level of detail used to classify time as during or outside work hours. We used scheduled patient visits (and physician schedules by extension) to define the EHR work outside work hours for each physician. We also included 30 minutes before and after each scheduled clinic time to capture preparatory and closing actions. Thus, we are confident that our time segment classifications truly reflect whether a physician was actively seeing patients or completing related tasks. Using clock time to define work versus after-work time might not always capture exactly when a physician starts and ends their actual workday.

The daily *time spent in the EHR* reported in this study (ie, 5 hours) is comparable with current estimates in the literature, which range from 1.5 to 5 hours [1,6,8,13,15,18,22]. With respect to the time spent per action category, most of the pediatricians’ time was spent *reviewing data and reports* both during and outside scheduled clinic hours. Only a small fraction of their time was spent completing *documentation and order entry actions.* This finding differs from reports in the literature and anecdotal evidence that indicate physicians spend most of their time completing documentation-related activities, particularly outside work hours [1-6,8,13,15,23,24]. For instance, the study by Overhage and Johnson [21] found that among their sample of pediatricians practicing in US-based ambulatory practices, documentation accounted for 31% of EHR use time, and chart review accounted for another 31% of EHR use time. The study by Arndt et al [8] found that nonteaching ambulatory physicians spent 44% of the total EHR use time engaged in clerical and administrative tasks (eg, documentation, order entry, billing and coding, and system security). Another study by Tai-Seale et al [15] found that primary care physicians spent 51% of their time completing EHR work, and 34% of this portion was spent on progress notes. A more recent study by Holmgren et al [13] found that ambulatory clinicians in the United States spent 67% of their EHR use time completing notes and orders.

One of the reasons why documentation time estimates in this study were lower than those commonly reported in the literature may be the differences in the categorization of EHR actions. In the abovementioned studies, the time spent viewing patient data during the process of writing a progress note may not have been distinguished from the total time spent on the note (ie, from the time the note was opened until it was finally signed). In this study, raw access log data were used to categorize each EHR action into one of the EHR action categories. No meanings were inferred—all viewing actions were categorized under *reviewing data and reports*, whereas all data entry actions were categorized as *documentation*. The level of granularity and objectivity used in this study ensures the robustness of our categorization and estimates. Another reason for the relatively lower documentation time estimates may be attributed to the extensive use of documentation templates with quick selection options and prepopulated data, as well as the extensive use of outpatient order sets for good childcare and common presenting complaints at this hospital. These practices may contribute to the reduced time spent on the EHR on documentation activities.

In addition, we found that pediatricians spend approximately 10% of their time completing *inbox and communication actions* both during and outside scheduled clinic hours. This estimate is lower than that reported in prior studies. The study by Holmgren et al [13] found that inbox activities accounted for approximately 14% of EHR work, whereas Arndt et al [8] and Tai-Seale et al [15] reported 24% and 22%, respectively. Interestingly, in this study, the loading and viewing of inbox messages were the most frequent and longest EHR actions outside of scheduled clinic hours in the *communication* category; however, during scheduled clinic hours, the most frequent and longest EHR action was the creation of messages. Perhaps physicians spend time checking their messages outside scheduled clinic hours to stay abreast with current patient needs but wait to respond to these messages during their scheduled clinic hours. A second study by Tai-Seale et al [25] found that receiving an excessive amount of system-generated inbox messages was associated with a higher probability of burnout and intention to reduce clinical work time, suggesting that this aspect of EHR work can have considerable effects on a physician’s well-being. On the other hand, a more recent study by Melnick et al [26] suggested that EHR inbox management was associated with physician departure—less time spent on EHRs was associated with physician departure. Although their finding was counterintuitive, they proposed that tracking EHR metrics could potentially identify physicians at a high risk of departure [26].

### Factors Associated With EHR Use

This study found no association of age, sex, or cFTE with EHR use. This finding is in contrast to previous findings from this research group [27]. In our previous work, we found that female physicians spend more time than male physicians using the EHR during work hours but not outside work hours. Provider-to-provider variation was the largest and most dominant source of variation in EHR use outside work hours, accounting for 52% of the total variance. However, in that study, EHR work outside work hours was defined using clock time, whereas our approach in this study of using actual physician schedules may have produced more accurate estimations, which could have eliminated bias in our prior models that accounted for the observed differences.

### EHR Access Log Data Use in Research

The use of EHR access logs is valid for assessing EHR actions as there is consistency between the direct observation findings, physician self-reported EHR work outside work hours, and EHR system event log data [8,28]. Although EHR access log data are highly complex, often uncharacterized, and require powerful statistical software and technical skills for processing and analysis, understanding and using raw EHR data could serve as an external validation of EHR vendor–supplied metrics. This validation is important as many researchers and hospital administrators use vendor-supplied data to explore research questions because of their ready availability and ease of use. However, the proprietary algorithms used by EHR vendors (eg, Epic’s Signal and Cerner’s LightsOn) use a *black box* methodology, wherein the actual composition of each metric is unknown. This study remedies this limitation by exposing and using raw access log data to produce more meaningful metrics and analyses.

At present, there are no agreed-upon standards for categorizing EHR actions. A standard categorization scheme for EHR actions will help provide a common language to facilitate and promote clear communication in this nascent research space. Upon close review of action categories used in the abovementioned studies, other studies in the scientific literature, and this study, the following *conceptual* categorization scheme of clinical EHR actions seems adequate as a foundation on which to further build: data review, data entry, data transmission, and other (Table 5). Rather than creating new action categories with each new research study, we propose building on the aforementioned categories, as this classification scheme is both clear and clinically meaningful. As it relates to this study, the proposed conceptual classification scheme aligns well with the categories used in this study in that data review aligns with *reviewing data and records*, data entry aligns with *creating and authenticating documentation* and *entering and authenticating orders*, data transmission aligns with *completing inbox and communication tasks*, and *other* aligns with *log-in and logout activities*.

The set of EHR action categories used by Zheng et al [29] (ie, reading, entering, printing, processing, log-in, and logout) is arguably one of the clearest among the available classifications used in the literature as it objectively categorizes the action without assigning any meaning—for instance, reading versus chart review. Perhaps, this strength is also the reason researchers refrain from using it; that is, the categories lack clinical meaning. The action categories used by Arndt et al [8] (ie, medical care, clerical, and inbox) have the opposite issue: they have clinical meaning but are somewhat ambiguous. For instance, some of the actions in the category of *medical care* could also be seen as clerical tasks. The categories used by Holmgren et al [13] closely align with those used in this study, are clear, and have clinical meaning.

**Table 5:** Proposed conceptual EHR^a^ action categorization scheme.

Conceptual EHR action categorization scheme	Action categories used in this study	Action categories used by Holmgren et al [13]	Action categories used by Arndt et al [8]	Action categories used by Zheng et al [29]
Data review—information review, retrieval, or gathering activities	Reviewing data and reports	Clinical review	Medical care	Reading
Data entry—information entry or recording activities	Creating and authenticating documentation; entering and authenticating orders	Notes; orders	Clerical	Entering
Data transmission—information transmission activities	Inbox and communication tasks	In-basket messages	Inbox	Printing
Other—other nonclinical activities	Log-in and logout activities	N/A^b^	N/A	Log-in, logout, and processing

^a^EHR: electronic health record.

^b^N/A: not applicable.

### Strengths and Limitations

A major strength of this study is the level of objectivity and granularity used to define time segments and action categories. Time segments were defined using actual scheduled patient visits to construct the physician workday schedules. These schedules were validated against the planned physician schedules. To the best of our knowledge, no study has used actual schedules to define EHR outside of work hours. In addition, action categories were defined using clear and objective criteria. Such a concise categorization of EHR work outside work hours and EHR action categories facilitates more accurate estimations. However, there are a few limitations to this study.

First, we were unable to parse chart review actions associated with other action categories. Several chart review actions are associated with other action categories. For instance, documentation-related actions are usually associated with chart review actions, and the methodology used in this study did not capture these nuanced associations. For example, if a physician viewed previous clinical notes while writing their own clinical note for that encounter, this action was classified as *reviewing data and reports*; however, to the physician, this viewing action might be more cognitively associated with documentation. This may explain why our estimates for the *reviewing data and reports* category were relatively high. This explanation also addresses why we found that physicians in this study spent only a small fraction of their time completing documentation and order entry actions, which was lower than the estimates in the literature and anecdotal evidence. The current scientific literature suggests that physicians spend a considerable amount of time outside work hours completing documentation-related activities [8], although the EHR is purported to contribute to more efficient use of physicians’ time.

In addition, this study did not have (and therefore did not include) work RVU (wRVU) as a factor in the regression analysis. wRVU is a key factor for understanding EHR work. Typically, wRVU indicates the volume and intensity of medical services provided; thus, the higher the wRVU, the more likely it is for a physician to spend time with the EHR. The absence of wRVU in the regression models may be the reason they did not generate statistically significant associations. However, the findings are important as they provide a general characterization of EHR use by pediatricians at this institution.

Finally, the study sample size was limited to 1 calendar month of EHR activity data for a single practice, setting, type of provider, and commercial EHR system (ie, academic primary care pediatricians at NCH using the Epic system), thus limiting the generalizability of the study findings. For instance, our study findings may not be generalizable to other specialties, including primary care specialties for adults, nor are they likely generalizable to subspecialty academic practices—they are most relevant to the academic pediatric practice. Furthermore, EHR interfaces are often modified according to the needs of each provider system [22]. Thus, the reported EHR use statistics may not be generalizable to other institutions and provider groups. On the other hand, Epic’s EHR is the most widely used EHR in the United States and includes use metrics [30], making our findings widely comparable with other institutions and provider groups. Furthermore, the pediatric population is an important one, and the sample group (ie, primary care physicians) helps reduce the technical complexity of studying work during and outside work hours.

### Implications and Future Research

Primary care pediatricians care for many children during half-day sessions (often simultaneously), work with nurses and other support staff, and interact with patients at multiple points in their daily workflow [28]. In addition, they spend considerable time on EHRs during and outside work hours to document and provide care. Thus, there is a need to improve physician-computer interactions by streamlining EHR workflows [22]. These improvements will likely need to be customized so that they are relevant to the specific type of practice: general pediatrics, subspecialty pediatrics, and many variations of adult practices. To identify interventions to improve EHR design and use, physicians’ EHR actions must be properly characterized to better understand their various activities and use patterns [22]. By identifying specific EHR actions that consistently dominate computer use across multiple providers, more targeted, data-driven approaches could be developed to improve physician-computer interactions [22]. This implication reinforces the need to validate proprietary algorithms and metrics generated by EHR vendors, as many researchers and hospital administrators rely on these metrics (vs computing them from raw EHR log data) for clinical, research, and policy purposes. This is understandable, given the tremendous complexity and resource requirements for working with and processing raw EHR access log data.

Contrary to prior research and anecdotal evidence, our analysis found that pediatricians spend a moderate amount of time on EHRs outside of scheduled clinic hours and relatively less time completing documentation-related tasks. As described previously, this hospital uses documentation templates extensively, which potentially helps reduce documentation time in the EHR. Other medical facilities may consider adopting such usability features to reduce the documentation burden among providers. With the issue of EHR documentation burden being prevalent among physicians and contributing to burnout among this group [31-35], opportunities to reduce the burden may help enhance physician well-being.

There are many opportunities for future research in this area, including standardizing vendor-derived EHR data descriptions in a way that is clinically relevant and important [26], validating the use of EHR access log data across different settings, exploring the relationship between EHR action frequency and EHR action duration, examining the contribution of EHR use outside work hours to physician well-being, determining overestimation and underestimation margins of estimates, and developing a taxonomy of EHR use to further promote consistency and valid comparisons across organizations and research studies. Such research will help provide additional insights into EHR workflow issues and the effect of EHR work on physician well-being. Furthermore, researchers in this field should strive to set standards [36,37], as we have proposed above. Accepted standards, for instance, on how to calculate work outside work hours and categorize EHR actions, will help facilitate research in this space.

### Conclusions

In this study, we used EHR access log data to identify actions typically completed outside scheduled clinic hours and the pattern of this EHR work. This study fills a gap in the literature by quantifying the use of EHR outside of scheduled clinic hours using actual scheduled patient visits rather than planned physician schedules or predefined clock times as a proxy. The findings from this study suggest that primary care pediatricians spend more than one-tenth of their EHR use time outside of scheduled clinic hours and that approximately three-quarters of this time is spent reviewing data and reports, whereas negligible time is spent completing orders. Further studies are needed to explore EHR use patterns by physicians and the reasons for these patterns to help improve EHR work and workflow. Qualitative and mixed methods research studies will be instrumental in gaining insights into these patterns.

## Abbreviations

cFTE: clinical full-time equivalent
EHR: electronic health record
FTE: full-time equivalent
NCH: Nationwide Children’s Hospital
RVU: relative value unit
wRVU: work relative value unit

## References

[1] Fletcher KE, Visotcky AM, Slagle JM, Tarima S, Weinger MB, Schapira MM (2012). The composition of intern work while on call. J Gen Intern Med.

[2] Oxentenko AS, Manohar CU, McCoy CP, Bighorse WK, McDonald FS, Kolars JC, Levine JA (2012). Internal medicine residents' computer use in the inpatient setting. J Grad Med Educ.

[3] Carayon P, Wetterneck TB, Alyousef B, Brown RL, Cartmill RS, McGuire K, Hoonakker PL, Slagle J, Van Roy KS, Walker JM, Weinger MB, Xie A, Wood KE (2015). Impact of electronic health record technology on the work and workflow of physicians in the intensive care unit. Int J Med Inform.

[4] Oxentenko AS, West CP, Popkave C, Weinberger SE, Kolars JC (2010). Time spent on clinical documentation: a survey of internal medicine residents and program directors. Arch Intern Med.

[5] Sinsky C, Colligan L, Li L, Prgomet M, Reynolds S, Goeders L, Westbrook J, Tutty M, Blike G (2016). Allocation of physician time in ambulatory practice: a time and motion study in 4 specialties. Ann Intern Med.

[6] Cox ML, Farjat AE, Risoli TJ, Peskoe S, Goldstein BA, Turner DA, Migaly J (2018). Documenting or operating: where is time spent in general surgery residency?. J Surg Educ.

[7] Wright AA, Katz IT (2018). Beyond burnout - redesigning care to restore meaning and sanity for physicians. N Engl J Med.

[8] Arndt BG, Beasley JW, Watkinson MD, Temte JL, Tuan WJ, Sinsky CA, Gilchrist VJ (2017). Tethered to the EHR: primary care physician workload assessment using ehr event log data and time-motion observations. Ann Fam Med.

[9] Poissant L, Pereira J, Tamblyn R, Kawasumi Y (2005). The impact of electronic health records on time efficiency of physicians and nurses: a systematic review. J Am Med Inform Assoc.

[10] Gregory ME, Russo E, Singh H (2017). Electronic health record alert-related workload as a predictor of burnout in primary care providers. Appl Clin Inform.

[11] Miyasaki JM, Rheaume C, Gulya L, Ellenstein A, Schwarz HB, Vidic TR, Shanafelt TD, Cascino TL, Keran CM, Busis NA (2017). Qualitative study of burnout, career satisfaction, and well-being among US neurologists in 2016. Neurology.

[12] Kroth PJ, Morioka-Douglas N, Veres S, Pollock K, Babbott S, Poplau S, Corrigan K, Linzer M (2018). The electronic elephant in the room: physicians and the electronic health record. JAMIA Open.

[13] Holmgren AJ, Downing NL, Bates DW, Shanafelt TD, Milstein A, Sharp CD, Cutler DM, Huckman RS, Schulman KA (2021). Assessment of electronic health record use between us and non-US health systems. JAMA Intern Med.

[14] Rotenstein LS, Holmgren AJ, Downing NL, Bates DW (2021). Differences in total and after-hours electronic health record time across ambulatory specialties. JAMA Intern Med.

[15] Tai-Seale M, Olson CW, Li J, Chan AS, Morikawa C, Durbin M, Wang W, Luft HS (2017). Electronic health record logs indicate that physicians split time evenly between seeing patients and desktop medicine. Health Aff (Millwood).

[16] Young RA, Burge SK, Kumar KA, Wilson JM, Ortiz DF (2018). A time-motion study of primary care physicians' work in the electronic health record era. Fam Med.

[17] Holmgren AJ, Lindeman B, Ford EW (2021). Resident physician experience and duration of electronic health record use. Appl Clin Inform.

[18] Rotenstein LS, Holmgren AJ, Downing NL, Longhurst CA, Bates DW (2021). Differences in clinician electronic health record use across adult and pediatric primary care specialties. JAMA Netw Open.

[19] Sinsky CA, Rule A, Cohen G, Arndt BG, Shanafelt TD, Sharp CD, Baxter SL, Tai-Seale M, Yan S, Chen Y, Adler-Milstein J, Hribar M (2020). Metrics for assessing physician activity using electronic health record log data. J Am Med Inform Assoc.

[20] Adler-Milstein J, Huckman RS (2013). The impact of electronic health record use on physician productivity. Am J Manag Care.

[21] Overhage JM, Johnson KB (2020). Pediatrician electronic health record time use for outpatient encounters. Pediatrics.

[22] Wang JK, Ouyang D, Hom J, Chi J, Chen JH (2019). Characterizing electronic health record usage patterns of inpatient medicine residents using event log data. PLoS One.

[23] Christino MA, Matson AP, Fischer SA, Reinert SE, Digiovanni CW, Fadale PD (2013). Paperwork versus patient care: a nationwide survey of residents' perceptions of clinical documentation requirements and patient care. J Grad Med Educ.

[24] Friedberg MW, Chen PG, Van Busum KR, Aunon F, Pham C, Caloyeras J, Mattke S, Pitchforth E, Quigley DD, Brook RH, Crosson FJ, Tutty M (2014). Factors affecting physician professional satisfaction and their implications for patient care, health systems, and health policy. Rand Health Q.

[25] Tai-Seale M, Dillon EC, Yang Y, Nordgren R, Steinberg RL, Nauenberg T, Lee TC, Meehan A, Li J, Chan AS, Frosch DL (2019). Physicians' well-being linked to in-basket messages generated by algorithms in electronic health records. Health Aff (Millwood).

[26] Melnick ER, Fong A, Nath B, Williams B, Ratwani RM, Goldstein R, O'Connell RT, Sinsky CA, Marchalik D, Mete M (2021). Analysis of electronic health record use and clinical productivity and their association with physician turnover. JAMA Netw Open.

[27] Attipoe S, Huang Y, Schweikhart S, Rust S, Hoffman J, Lin S (2019). Factors associated with electronic health record usage among primary care physicians after hours: retrospective cohort study. JMIR Hum Factors.

[28] Hribar MR, Read-Brown S, Goldstein IH, Reznick LG, Lombardi L, Parikh M, Chamberlain W, Chiang MF (2018). Secondary use of electronic health record data for clinical workflow analysis. J Am Med Inform Assoc.

[29] Zheng K, Ciemins EL, Lanham HJ, Lindberg C, Man D Examining the relationship between health it and ambulatory care workflow redesign - final report. Agency for Healthcare Research and Quality.

[30] Baxter SL, Apathy NC, Cross DA, Sinsky C, Hribar MR (2021). Measures of electronic health record use in outpatient settings across vendors. J Am Med Inform Assoc.

[31] Collier R (2017). Electronic health records contributing to physician burnout. CMAJ.

[32] Shanafelt TD, Dyrbye LN, Sinsky C, Hasan O, Satele D, Sloan J, West CP (2016). Relationship between clerical burden and characteristics of the electronic environment with physician burnout and professional satisfaction. Mayo Clin Proc.

[33] DiAngi YT, Longhurst CA, Payne TH (2016). Taming the EHR(electronic health record) - there is hope. J Fam Med.

[34] Harris DA, Haskell J, Cooper E, Crouse N, Gardner R (2018). Estimating the association between burnout and electronic health record-related stress among advanced practice registered nurses. Appl Nurs Res.

[35] Robinson KE, Kersey JA (2018). Novel electronic health record (EHR) education intervention in large healthcare organization improves quality, efficiency, time, and impact on burnout. Medicine (Baltimore).

[36] Rule A, Chiang MF, Hribar MR (2020). Using electronic health record audit logs to study clinical activity: a systematic review of aims, measures, and methods. J Am Med Inform Assoc.

[37] Adler-Milstein J, Zhao W, Willard-Grace R, Knox M, Grumbach K (2020). Electronic health records and burnout: time spent on the electronic health record after hours and message volume associated with exhaustion but not with cynicism among primary care clinicians. J Am Med Inform Assoc.

